# Self-perceived food literacy is positively associated with diet quality among Dutch individuals with type 2 diabetes

**DOI:** 10.1016/j.pmedr.2025.103229

**Published:** 2025-08-30

**Authors:** Iris van Damme, Eva R. van Veldhuisen, Johanna M. Geleijnse, Renate M. Winkels

**Affiliations:** Division of Human Nutrition and Health, Wageningen University, PO Box 17, 6700AA Wageningen, the Netherlands

**Keywords:** Nutrition, Diet, Type 2 diabetes, Food literacy, Diet quality

## Abstract

**Objective:**

This cross-sectional study was conducted to investigate associations between food literacy and diet quality among individuals with type 2 diabetes.

**Method:**

In the Netherlands in 2022/2023, 166 individuals with type 2 diabetes (66.8 ± 10.3 years, BMI 28.2 ± 4.3 kg/m^2^) completed questionnaires. Self-perceived food literacy (SPFL) was assessed using the 29-item SPFL-scale (score 1–5, higher scores indicating higher food literacy). Diet quality was assessed with a brief frequency questionnaire (FFQ; score 0–160, higher scores indicating higher diet quality). Associations between food literacy and diet quality were evaluated with linear models.

**Results:**

Mean diet quality score was 107 ± 16 and mean food literacy score was 3.7 ± 0.4. Linear regression showed that 1-point higher food literacy scores were associated with 14-point higher diet quality scores (β adjusted 14.0; 95 % CI: 8.4 to 19.6). Individuals with above-median food literacy scores had diet quality scores that were 12.5 points higher (95 % CI: 7.7 to 17.3) than individuals with below-median scores. Food preparation skills, healthy budgeting, social and conscious eating, resilience and resistance, and healthy snack styles, were associated with better diet quality.

**Conclusion:**

Associations between several food literacy domains and diet quality in people with type 2 diabetes indicate the importance of promoting food literacy in this group.

## Highlights

• A healthy diet plays a crucial role in management of type 2 diabetes.
• Food literacy is associated with diet quality in adults with type 2 diabetes.
• Several domains of food literacy are associated with diet quality.
• Promoting food literacy is important to improve diet quality in diabetes patients.

## 1 Introduction

Type 2 diabetes (T2D) is an important cause of global morbidity and mortality, with rising economic and societal consequences (Sun et al., 2022). In diabetes management, diet therapy plays a crucial role since diet is a modifiable lifestyle factor that is associated with glycemic control and cardiovascular outcomes. Dietary guidelines recommend consuming a high-quality diet (Evert et al., 2019), but improving diet quality is challenging since changing dietary intake requires a behavior change that is not easily made and sustained (Salvia and Quatromoni, 2023). Consequently, diet quality remains suboptimal in many individuals with diabetes(Sun et al., 2017).

Changing healthy dietary behavior is difficult and requires knowledge, attitudes, and skills to use information that promotes a healthier diet. Individuals should be literate in meal planning, food preparation, label reading, and critically evaluating nutrition information to make healthy choices in a variety of situations (Fernandez et al., 2019). These essential domains for changing dietary behavior are collectively called ‘food literacy’ and could be the key to improving dietary interventions for T2D (Velardo, 2015; Poelman et al., 2018; Thompson et al., 2021).

Although previous studies have established a general association between food literacy and diet quality, there is limited evidence specifically among adults with T2D. Furthermore, few studies have examined how distinct components of food literacy relate to dietary patterns in this population. A recent systematic review on studies in people with T2D concluded that interventions targeting nutrition education improved patient engagement (Savarese et al., 2021), but the impact on diet quality was not investigated. A cross-sectional study among 288 Iranian adults with T2D indicated that individuals with higher food literacy scores had higher intakes of vegetables, fruits, and better diet quality overall, but individual dimensions of food literacy were not assessed (Bastami et al., 2023).

To target dietary interventions more precisely to the needs of people with T2D, more insight is needed into the relationship between individual food literacy items and dietary patterns. The main aim of this study is to explore the dietary behavior of adults with T2D and specifically the associations between various dimensions of food literacy and diet quality. Insight into these relationships may provide useful information for improvements in dietary counseling and future dietary intervention studies.

## 2 Methods

### 2.1 Study design

This study was conducted on data from 166 people with T2D living in the Netherlands. A digital survey was designed using Qualtrics software to collect data on food literacy and diet quality. To increase sample size and improve the robustness of our results, we combined data from this online survey with baseline data from a previously conducted randomized controlled trial (RCT). Populations, variables, and questionnaires, were comparable, allowing for pooled cross-sectional analysis. No longitudinal or intervention-related data from the RCT were used.

Participants for the online survey study were recruited between January 2022 and December 2023 via volunteer databases of the university, flyers in supermarkets and dieticians' offices, and websites of patient organizations. Adults (≥ 18 years of age) diagnosed with T2D were eligible for participation. Respondents provided digital consent at the start of the survey. The survey was exempt from review by a medical ethical committee as it was not subjected to the Medical Research Involving Human Subjects Act (In Dutch: WMO).

The 6-month RCT compared a dietary counseling intervention with no intervention (control) in adults with a previous diagnosis of non-insulin-dependent T2D who were not receiving current dietary counseling from a dietitian. Adults (≥ 18 years of age) with a diabetes duration of at least six months were recruited from October 2022 to December 2023 by advertisement through flyers, social media, email lists, patient organizations, word of mouth, and snowballing recruitment methods. Details on the trial are described elsewhere (van Damme et al., 2025). The study was approved by the Medical Ethical Committee of East Netherlands (reference NL80697.091.22) and was performed in accordance with the Declaration of Helsinki. All participants provided written informed consent prior to enrolment. The trial was registered at clinicaltrials.gov as NCT05666843.

### 2.2 Measures and variables

Participants from both cohorts received online questionnaires to collect information on food literacy, diet quality, and demographics. Trial participants received these questionnaires before they visited the research facility for measurements.

#### 2.2.1 Food literacy

All participants completed the Self-Perceived Food Literacy (SPFL) scale (Poelman et al., 2018) to assess food literacy. The SPFL scale is an expert-based and theory-driven tool that consists of 29 items related to eight domains of food literacy, which are food preparation skills (six items), resilience and resistance (six items), healthy snack styles (four items), social and conscious eating (three items), examining food labels (two items), daily food planning (two items), healthy budgeting (two items), and healthy food stockpiling (four items). Respondents answered the 29 questions on a five-point Likert scale, ranging from ‘no/never’ to ‘yes/always’. Scores per domain are calculated as the sum of the scores divided by the number of items for the domain, giving a score between one and five per domain. The total food literacy score is calculated as the average of all 29 questions, and ranges from one to five, with higher scores reflecting higher levels of food literacy.

#### 2.2.2 Diet quality

Diet quality was assessed with an online brief Food Frequency Questionnaire (Eetscore FFQ) (Looman et al., 2017). The Eetscore FFQ is a screener of diet quality, assessing adherence to the Dutch dietary guidelines by calculating Dutch Healthy Diet Index (DHD15-index) scores. The DHD15-index consists of 16 components representing the Dutch dietary guidelines: vegetables, fruit, wholegrain products, legumes, nuts, dairy products, fish, tea, fats and oils, coffee, red meat, processed meat, sweetened beverages and fruit juices, alcohol, salt, and unhealthy choices. For all components, a maximum of 10 points can be obtained, resulting in a total score of 0 to 160 points, with higher scores reflecting better adherence to the guidelines. More information about the DHD15-index is described elsewhere (De Rijk et al., 2022).

#### 2.2.3 Other variables

Data on age, self-identified gender, education, household size, postal code, ethnicity, and diabetes treatment (no medication, oral medication, insulin, or a combination) were collected via online questionnaires. Participants from the survey study self-reported body weight and height. For trial participants, anthropometric data were measured twice at baseline and averaged. Height was measured without shoes on a wall-mounted stadiometer, and weight was measured in light indoor clothing without shoes.

For all individuals, postal codes at time of enrollment were used to estimate Socio-Economic Position (SEP). SEP scores are provided by Statistics Netherlands (CBS) for each of the 4059 four-digit postal code areas in the Netherlands for the year 2021 and based on (1) mean annual income per household, (2) percentage of households with a low income, and (3) percentage of households with a low education. SEP was divided into three groups: low (1st-3rd deciles, 1187 postal codes), intermediate (4th–7th, 1200 postal codes), and high (8th–10th, 1189 postal codes) SEP tertile.

### 2.3 Statistical analysis

Data from both cohorts were pooled, and individuals were categorized into two groups based on their food literacy score (below or above the median food literacy score) to identify differences in characteristics and DHD15-index scores. Student's *t*-tests were used to compare continuous variables, Fisher's exact test for categorical variables with two groups (gender, household size), and analysis of variance for categorical variables with ≥ three groups (education, SEP tertile, and diabetes medication). As a follow-up to the analysis of variance models, pairwise comparisons using univariate F-tests were used to evaluate statistically significant differences.

To examine the association between the total food literacy score and the diet quality score, univariate linear regression was used. Similarly, we examined the associations of the eight food literacy domain scores with the diet quality score individually. Regression coefficients (β) with 95 % CIs were calculated to provide the difference in the DHD15-index score per one-point increase in the SPFL total score. Models were adjusted for age, gender, body mass index (BMI), and SEP tertile, as these factors are known to be associated with literacy and diet quality (Murakami et al., 2024). Additionally, medication use was added as a potentially confounding factor as the percentage of individuals using no diabetes medication differed between individuals with high and low food literacy.

Data analyses were performed with R® version 4.4. For continuous variables, mean ± standard deviation, or median and interquartile range are presented, as applicable. Proportions are presented for categorical data.

## 3 Results

**Fig. 1:**
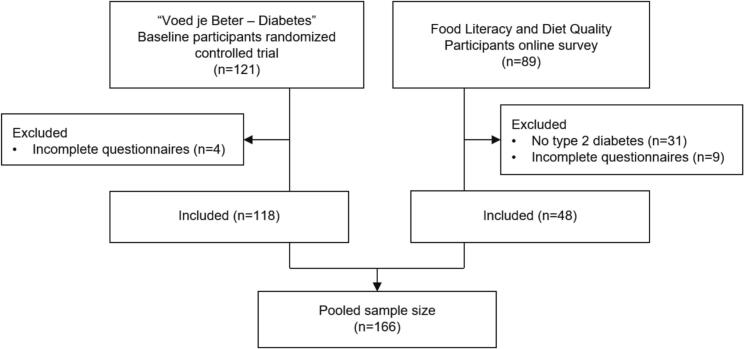
Flow chart of the study population selection.

A total of 48 individuals completed the survey, and 118 trial participants completed the questionnaires (Fig. 1). The mean age of all 166 individuals was 66.8 ± 10.3 years, the majority were male (*n* = 105, 63 %) and 55 % completed higher education. Average diet quality score was 107 ± 16.5 out of 160, and average food literacy score 3.7 ± 0.4 out of 5 (Table 1).

**Table 1:** Demographic and anthropometric characteristics according to food literacy of 166 individuals with type 2 diabetes in the Netherlands, 2023.

Characteristics	Total (*n* = 166)	Food literacy level according to SPFL-scale a	
		Lower (*n* = 80)	Higher (*n* = 86)
Age (y)	66.8 ± 10.3	65.6 ± 10.8	67.9 ± 9.76
Women (n, %) ⁎	61 (36.7)	22 (27.5)	39 (45.3)
Education (n, %)			
Low	12 (7.2)	6 (7.5)	6 (7.0)
Medium	62 (37.3)	33 (41.3)	29 (33.7)
High	92 (55.4)	41 (51.3)	51 (59.3)
Body weight (kg) ⁎	86.7 ± 15.8	89.2 ± 15.7	84.3 ± 15.7
BMI (kg/m^2^)	28.2 ± 4.3	28.5 ± 4.3	27.9 ± 4.2
Living alone (n, %)	36 (21.7)	18 (22.5)	18 (20.9)
SEP Tertile (n, %)			
Low	58 (34.9)	31 (38.8)	27 (31.4)
Medium	63 (38)	25 (31.3)	38 (44.2)
High	45 (27.1)	24 (30)	21 (24.4)
Diabetes medication use (n, %)			
No medication	21 (12.7)	5 (6.3)	16 (18.6)
Oral medication	136 (81.9)	70 (87.5)	66 (76.7)
Insulin	9 (5.4)	5 (6.3)	4 (4.7)
Diabetes duration in years	10.8 ± 8.6	12.3 ± 9.5	9.2 ± 7.2
Unknown	59 (35.5)	24 (30)	35 (40.7)
SPFL-scale ⁎	3.7 ± 0.4	3.3 ± 0.3	4.0 ± 0.2

Continuous values are presented as mean ± standard deviation.

a SPFL = Self-Perceived Food Literacy. The median SPFL score of 3.7 was used to discriminate between lower vs higher SPFL.

⁎ Significant difference in values for lower vs higher food literacy scores (*p* < 0.05).

Individuals were categorized into two groups based on their food literacy scores: those with a score below the median (*n* = 80) or the median and above (*n* = 86). The group with higher food literacy scores had a higher proportion of women (45 %) compared to the group with lower food literacy scores (28 %). Although body weight differed significantly between individuals with lower and higher food literacy, no significant difference was observed in BMI (Table 1). Diabetes medication use was more prevalent among the individuals with lower food literacy scores, with 94 % of patients with lower food literacy scores using diabetes medication compared to 81 % of patients with higher food literacy scores. Average diabetes duration, if known, was higher in the group with lower food literacy scores compared to the group with higher food literacy scores.

Diet quality scores were 12.5 points higher (95 % CI: 7.7 to 17.3) in the group with higher food literacy compared to the group with lower food literacy. Individuals with higher food literacy scores scored significantly higher on adherence to guidelines for vegetables, fruit, legumes, fish, processed meat, sweetened beverages, and fruit juices, and unhealthy choices (Table 2).

**Table 2:** Mean diet quality scores for different dietary components according to food literacy of 166 patients with type 2 diabetes in the Netherlands, 2023.

Diet quality scores (DHD15-Index)		Food literacy level according to SPFL-scale^a^		
	Total (*n* = 166)	Lower (*n* = 80)	Higher (*n* = 86)	Difference (95 % CI)
Total score	106.8 ± 16.5	100.2 ± 14.5	112.7 ± 16.1	12.5 (7.7; 17.3)
Component scores				
Vegetables	6.1 ± 3.0	5.2 ± 2.9	7.0 ± 2.8	1.8 (0.9; 2.7)
Fruit	6.6 ± 3.1	5.7 ± 3.2	7.4 ± 2.6	1.7 (0.8; 2.6)
Wholegrain products	7.5 ± 2.8	7.5 ± 2.8	7.6 ± 2.8	0.1 (−0.8; 0.9)
Legumes	7.0 ± 3.9	6.2 ± 4.1	7.7 ± 3.5	1.4 (0.3; 2.6)
Nuts	6.3 ± 3.7	6.0 ± 3.7	6.6 ± 3.6	0.6 (−0.5; 1.7)
Dairy products	5.9 ± 3.1	6.0 ± 3.2	5.8 ± 2.9	−0.2 (−1.2; 0.7)
Fish	6.7 ± 3.3	6.0 ± 3.2	7.4 ± 3.2	1.4 (0.4; 2.4)
Tea	4.2 ± 4.1	3.7 ± 3.8	4.6 ± 4.3	1.0 (−0.3; 2.2)
Fat and oils	5.0 ± 4.7	5.3 ± 4.6	4.8 ± 4.7	−0.5 (−2.0; 0.9)
Coffee	6.6 ± 2.8	6.9 ± 2.7	6.4 ± 2.9	−0.5 (−1.3; 0.4)
Red meat	8.9 ± 2.6	9.1 ± 2.4	8.7 ± 2.7	−0.4 (−1.2; 0.4)
Processed meat	4.7 ± 3.6	4.1 ± 3.6	5.3 ± 3.6	1.2 (0.1; 2.3)
Sweetened beverages and fruit juices	8.7 ± 2.6	8.0 ± 3.1	9.3 ± 1.8	1.2 (0.5; 2.0)
Alcohol	8.9 ± 2.6	8.6 ± 2.8	9.1 ± 2.4	0.5 (−0.4; 1.3)
Salt	8.6 ± 1.8	8.5 ± 1.7	8.6 ± 1.9	0.1 (−0.5; 0.6)
Unhealthy choices	5.2 ± 4.2	3.6 ± 4.0	6.6 ± 3.8	3.1 (1.9; 4.3)

Values are presented as mean ± standard deviation or regression coefficient B (95 % CI).

DHD15-index: Dutch Healthy Diet 2015 index. Higher scores indicate better adherence to the dietary guideline.

a SPFL = Self-Perceived Food Literacy. The median SPFL score of 3.7 was used to discriminate between lower vs higher SPFL.

Linear regression showed that 1-point higher food literacy scores were associated with 14-point higher diet quality scores (β adjusted 14.0; 95 % CI: 8.4 to 19.6, Table 3). Of the eight food literacy domains that were tested, food preparation skills, healthy budgeting, social and conscious eating, resilience and resistance, and healthy snack styles were significantly associated with higher diet quality scores. The domains examining food labels, healthy food stockpiling, and food planning were not associated with diet quality.

**Table 3:** Associations between self-perceived food literacy with total DHD15-index in 166 patients with type 2 diabetes in the Netherlands, 2023.

	Unadjusted model	Adjusted model a
SPFL total score	15.6 (10.0; 21.3)	14.0 (8.4; 19.6)
SPFL domain scores		
Examining food labels	1.2 (0.0; 2.3)	1.1 (−0.1; 2.3)
Food preparation skills	0.7 (0.2; 1.3)	0.6 (0.0; 1.1)
Healthy food stockpiling	0.4 (−0.4; 1.2)	0.4 (−0.4; 1.1)
Food planning	0.7 (−0.5; 1.9)	0.5 (−0.7; 1.7)
Healthy budgeting	3.9 (2.2; 5.7)	3.4 (1.6; 5.1)
Social and conscious eating	1.2 (0.1; 2.4)	1.4 (0.3; 2.5)
Resilience and resistance	1.9 (1.2; 2.6)	1.7 (0.9; 2.5)
Healthy snack styles	2.1 (1.3; 2.9)	1.9 (1.2; 2.7)

Values are presented as regression coefficient B (95 % CI).

SPFL = Self-Perceived Food Literacy.

a Adjusted for age, gender, BMI, SEP tertile and diabetes medication.

## 4 Discussion

In this study among 166 individuals with T2D, higher food literacy scores were associated with higher diet quality scores. Higher food literacy scores were more prevalent among women and among individuals who managed their diabetes without medication. Especially for the domains of food preparation skills, healthy budgeting, social and conscious eating, resilience and resistance, and healthy snack styles, higher scores were associated with higher diet quality in this population.

As food literacy is a relatively new concept (Thompson et al., 2021), few studies have investigated aspects of food literacy in populations with T2D. However, our finding that higher food literacy scores are associated with higher diet quality scores is consistent with previous studies in other populations. A systematic review including thirteen studies on food literacy and adolescents' dietary intake indicated that adolescents with greater food knowledge had healthier dietary practices (Vaitkeviciute et al., 2015). In a general Dutch adult population, higher levels of food literacy were associated with a significantly higher fruit, vegetable, and fish consumption (Poelman et al., 2018). In a cross-sectional study among 288 Iranian individuals with T2D, a positive association between food literacy and diet quality was found as well (Bastami et al., 2023).

In our study, five of the eight domains of food literacy were associated with diet quality: food preparation skills, healthy budgeting, social and conscious eating, resilience and resistance, and healthy snack styles. This suggests that there is not one single food literacy domain that strongly determines diet quality and that a person needs to be skilled in multiple domains to improve diet quality. Healthy dietary behavior involves decisions related to the selection, preparation, and consumption of food. People should be able to choose healthy options, be skilled to prepare them, and their budget must allow them to make those choices (Darmon and Drewnowski, 2015). The findings of our study also indicate that providing tools to cope with social and conscious eating, targeting resilience to unhealthy food choices, may be a suitable strategy to improve the diet quality of individuals with T2D (Luthar et al., 2000; Pesantes et al., 2015; O'Hearn, Lara-Castor et al., 2023; van Damme et al., 2025). Therefore, nutritional counseling for T2D should not only focus on increasing knowledge about healthy diets, but also support individuals in applying that knowledge through practical skills and self-management strategies. Given that focusing on isolated components, such as mindful eating, has shown limited and inconsistent effects on diet quality (Grider et al., 2021), we recommend targeting a broader range of food literacy domains to help people make sustainable improvements in their diet quality.

The domains examining food labels, food planning, and healthy food stockpiling were not associated with diet quality in our study. This may reflect either limited relevance for diet quality in people with T2D or shortcomings in how these domains were measured. For example, when food purchasing is done by another household member, literacy domains related to food purchases may not reflect the respondent's decision-making autonomy. Future interventions should consider involving household members to also get insight in habits of household members that influence an individual's dietary intake.

Self-perceived food literacy scores were relatively high in our study population. In a previous study in the Netherlands that applied the same food literacy questionnaire, an average food literacy score of 3.4 ± 0.5 was reported, which is lower than the 3.7 ± 0.4 in our study (Poelman et al., 2018). The higher levels of food literacy may arise from the fact that most individuals in our study completed some sort of higher education, which has previously been found to be associated with food literacy level (Poelman et al., 2018). Similarly, the diet quality scores in our study were relatively high when compared to other cohorts with individuals with T2D (Gal et al., 2024). This suggests that we included a group of highly educated individuals with T2D who were aware of what constitutes a healthy diet, which may not necessarily be representative of the broader population of individuals with diabetes. This limits the generalizability of our findings, groups with lower education levels or less access to health care. In these groups, even if people have good food literacy, it may be harder to put this knowledge into practice due to factors such as financial stress, a less supportive food environment, or fewer resources (Pechey and Monsivais, 2016). Future research should include more diverse groups to better understand how food literacy works in different parts of the population.

We observed that in our population, women showed higher food literacy scores compared to men, in line with previous studies on gender differences (Svendsen et al., 2021). Women cook more often, are more involved in weight control, and have stronger beliefs about healthy eating than men (Poelman et al., 2018), potentially explaining their higher food literacy scores. We also found that individuals with lower food literacy had higher body weight, while BMI hardly differed between groups. This discrepancy is likely due to gender differences, as the lower food literacy group included more men, who generally have higher body weight and height.

Surprisingly, we observed no clear differences in educational level between participants with a food literacy score below or above the median, which we attribute to the small number of individuals with low education level in our population. Individuals with higher food literacy levels in our study more often managed their diabetes without medication, possibly because these individuals are skilled enough to manage their glycemic blood values through nutrition alone, reflecting higher food literacy.

Our study design allowed for a quick and affordable way to gain insights into the associations between food literacy and diet quality in individuals with T2D, but it should be noted that food choices are not only influenced by the level of food literacy. Physiological needs, nutritional behavior of peers, social norms, availability of foods, personal experiences, and food preferences may also affect diet quality (Fernqvist et al., 2024). Additionally, we did not assess whether survey participants were receiving dietary counseling at the time of data collection, limiting our ability to evaluate its influence. Also, more detailed information on medication use could have provided additional insight on how diseases severity influences food literacy and diet quality. Furthermore, the cross-sectional design of our study did not allow us to research causal relationships or the direction of the relationship. It should also be acknowledged that pooling the data from two different cohorts may have resulted in a heterogeneous study population. While combining these datasets increased the sample size, our results still rely on a relatively small overall sample. Although we adjusted our models for a number of confounders, unmeasured or unknown factors could still have influenced our results.

The strengths of the present study include the use of well-established scales for assessing food literacy and diet quality. The Eetscore FFQ is, however, known to underestimate red meat and salt intake, and the high scores for these components may not necessarily reflect optimal intake levels (De Rijk et al., 2022). Although adherence scores for these components are comparable to those of other patient populations in the Netherlands (Heusschen et al., 2022; Lamers et al., 2023), dietary biomarkers or detailed assessment methods would be needed to accurately determine absolute nutrient intake. Another limitation of our study is the partial overlap with the COVID-19 pandemic, which may have affected associations between food literacy and diet quality scores. Previous research indicates that during the pandemic, people became more concerned about their health and well-being and dedicated more time and resources to cooking and eating, resulting in safer food choices of higher quality (Gianni et al., 2023) and possibly increased food literacy levels. Future studies should incorporate multiple timepoints and the assessment of clinical parameters such as glucose or lipid values to investigate the relation between food literacy, diet quality, and health.

## 5 Conclusion

To conclude, in this cross-sectional study among Dutch individuals with T2D, better food literacy was associated with higher diet quality. This underscores the importance of integrating food literacy in future research and public health settings to promote healthy dietary behavior. Future research could examine the effects of interventions that improve overall food literacy, with emphasis on literacy in food preparation skills, healthy budgeting, social and conscious eating, resilience and resistance, and healthy snack styles, to investigate whether improving these food literacy skills are effective strategies to improve diet quality and health outcomes of individuals with T2D.

## CRediT authorship contribution statement

**Iris van Damme:** Writing – original draft, Visualization, Project administration, Investigation, Formal analysis, Data curation, Conceptualization. **Eva R. van Veldhuisen:** Writing – review & editing, Project administration, Data curation. **Johanna M. Geleijnse:** Writing – review & editing, Supervision, Funding acquisition, Conceptualization. **Renate M. Winkels:** Writing – review & editing, Supervision, Conceptualization.

## Funding sources

The study is part of the Voed je Beter project, and this work was supported by Regio Deal Foodvalley (grant number 162135). The funders have no role in the study design; in the collection, analysis, and interpretation of data; in the writing of the report; or in the decision to submit the article for publication.

## Declaration of competing interest

The authors declare the following financial interests/personal relationships which may be considered as potential competing interests: Iris van Damme, Eva R van Veldhuisen, Renate M Winkels and Johanna M Geleijnse report financial support was provided by Netherlands Ministry of Health Welfare and Sport/Regiodeal Foodvalley. Johanna Marianna Geleijnse reports a relationship with Wageningen University & Research that includes: funding grants. Johanna M. Geleijnse is vice president of the Health Council of the Netherlands.

## Data Availability

The data that has been used is confidential.
